# Effect of spent coffee grounds addition on the microstructure and quality characteristics of protein-fortified bread

**DOI:** 10.1016/j.fochx.2026.103796

**Published:** 2026-03-27

**Authors:** Daesik Jo, Woojin Na, Yujin Wang, Kwang-Geun Lee

**Affiliations:** Department of Food Science and Biotechnology, Dongguk University-Seoul, 32, Dongguk-ro, Ilsandong-gu, Goyang-si, Gyeonggi-do, 410-820, Republic of Korea

**Keywords:** Spent coffee grounds, Dietary fiber, Bread quality characteristics, Microstructure, Upcycling

## Abstract

This study aimed to develop bread fortified with dietary fiber and protein using spent coffee grounds (SCG) and soy protein isolate and to analyze the effects of SCG addition on bread quality and microstructure. The addition of SCG led to a significant reduction in both the volume and specific volume of bread. Scanning electron microscopy revealed the development of a denser and more irregular crumb structure, with smaller cells and thicker cell walls observed at higher SCG levels. Texture profile analysis showed increased hardness and chewiness in samples with higher SCG addition, reflecting reduced crumb porosity. A 15% addition of SCG led to 7.1%, 10.7%, and over 40-fold increases in crude protein, crude ash, and crude fiber content, respectively, compared to the control. Sensory evaluation showed that breads containing 1–5% SCG exhibited comparable acceptability to the control, with some panelists preferring the enhanced coffee aroma and flavor complexity.

## Highlights

• Spent coffee grounds (SCG) were incorporated (1–15%, w/w) into soy protein–fortified bread.
• Increasing SCG levels reduced bread volume and produced a denser, irregular crumb structure.
• SCG addition enhanced crude protein, ash, and fiber contents, with >40-fold increase in fiber at 15%.
• Sensory evaluation indicated that 3–5% SCG breads maintained consumer acceptability.
• SCG shows potential as a sustainable ingredient for nutritional and functional bakery applications.

## 1 Introduction

Spent coffee grounds (SCG) are the insoluble residues obtained after the extraction of roasted and ground coffee beans (*Coffea* spp.). The coffee industry generates more than 6 million tons of SCG annually. With the global increase in coffee consumption, the efficient and sustainable management of SCG has become an increasingly challenging task (Ahanchi et al., 2024; Andrade et al., 2022a; Pagett et al., 2023). The accumulation of SCG in landfills contributes to environmental pollution, such as greenhouse gas emissions and soil acidification, and represents a loss of a potentially valuable resource. Considering environmental and economic impacts, strategies for transforming SCG into valuable ingredients for food or other industries have become essential. Recently, extensive research has explored the diverse applications of SCG beyond the food industry. For instance, SCG-derived materials have shown great potential as photothermal materials for high-efficiency solar-driven energy generation (Shi et al., 2023; Zheng et al., 2025). Furthermore, modified SCG and SCG-based biochars are highly effective in environmental remediation, particularly for the adsorption and removal of heavy metals from wastewater (Cheng et al., 2024; Cho et al., 2023; Cui et al., 2025). Alongside these diverse industrial and environmental applications, the valorization of SCG as a functional ingredient in the food industry remains a highly attractive strategy. Although previous studies have investigated the incorporation of SCG into various bakery foods, these foods are still rarely found in commercial markets (Aguilar-Raymundo et al., 2019; Ali et al., 2018; Hussein et al., 2019; Severini et al., 2020). This gap between research and commercial practice suggests potential challenges in consumer acceptance and sensory quality.

Bread primarily consists of carbohydrates and contains insufficient amounts of dietary fiber and protein. To overcome these limitations, protein-fortified bread, which is bread enriched with additional protein sources, has been increasingly studied (Bouras et al., 2021; Pořízka et al., 2023; Xing et al., 2021). For instance, supplementation with soy protein isolate (SPI) has been shown to enhance the protein content and improve the nutritional profile of bread, particularly by complementing the limited amino acid composition of bread flour (Ivanovski et al., 2012; Obasi et al., 2023). SPI is a cost-effective protein source that contains beneficial phytochemicals, such as isoflavones, and has relatively low allergenic potential compared to other plant-based proteins (Ivanovski et al., 2012; Maryniak et al., 2022). SCG has garnered attention as a fiber-rich byproduct capable of increasing the total dietary fiber content in baked products (Ahanchi et al., 2024; Martinez-Saez et al., 2017). SCG exhibits strong antioxidant activity due to the presence of Maillard reaction products and phenolic compounds (Martinez-Saez et al., 2017; Murthy & Naidu, 2012). In addition, SCG is rich in diverse volatile compounds that can impart a desirable coffee aroma to food products (Andrade et al., 2022b). Although SPI and SCG have been individually incorporated into bread formulations, their combined effect on bread quality and nutritional value has not yet been investigated. Incorporating both SCG and SPI into bread formulations is not merely a waste-recycling approach, but a strategic nutritional intervention. While conventional bread lacks dietary fiber and bioactive compounds, SCG serves as a highly desirable functional ingredient that compensates for these nutritional deficiencies. By upcycling SCG, this formulation provides a unique combination of high dietary fiber, potent antioxidant capacity from phenolic compounds, and cost-effective protein fortification from SPI, all while imparting a naturally appealing coffee aroma without the need for synthetic flavorings. Therefore, this approach offers a meaningful innovation that simultaneously addresses environmental sustainability and the consumer demand for health-promoting functional bakery products.

Bread quality attributes, such as texture, appearance, and sensory acceptability, are closely related to its microstructural properties. Analyzing bread microstructure provides fundamental insights into the gluten-starch interactions, cell wall, and porosity, which collectively influence gas retention capacity and bread volume (Demirkesen et al., 2014). Structural characterization through microscopic techniques, including scanning electron microscopy (SEM) and light microscopy, enables detailed observation of changes induced by ingredient modifications. This allows researchers to precisely determine how ingredient interactions affect bread quality at the cellular level, thereby guiding the optimization of formulations and processing parameters. Therefore, microstructural analysis is essential for comprehensively understanding and improving bread quality, particularly when novel ingredients, such as dietary fiber or alternative protein sources, are incorporated into bread formulations.

For the commercial production of SCG- and SPI-enriched bread, it is essential to investigate the effects of SCG addition on bread characteristics. This study aimed to: (1) evaluate changes in bread quality characteristics, such as volume, color, proximate composition, and texture, resulting from the incorporation of SCG; (2) assess consumer acceptability through sensory evaluation; and (3) examine microstructural changes using SEM, while employing X-ray diffraction (XRD) to determine the effect of SCG on the relative crystallinity (RC) of the bread matrix.

## 2 Materials and methods

### 2.1 Materials

SCG was obtained from Urban Labs Inc. (Seoul, Korea), which processes SCG to reduce bitterness and remove caffeine for individuals sensitive to caffeine. The SCG was crushed using a mixer and sieved through a 355 μm mesh (Chunggyesanggongsa, Seoul, Korea) before use. The composition of SCG on a dry basis (g/100 g) was 63.89 carbohydrate, 39.37 fiber, 17.77 protein, 16.07 lipid, 1.42 moisture, and 0.85 ash. The proximate composition of SCG was determined in this study, while the nutritional information for bread flour, SPI and unsalted butter was obtained from manufacturer-provided data. Bread flour (Daehan Flour Mills Co., Ltd., Seoul, Korea; 73 g/100 g carbohydrate, 13.5 g/100 g moisture, 12 g/100 g protein, 1.4 g/100 g lipid, and 0.42 g/100 g ash), SPI (Jayoncho, Seoul, Korea; 91.2 g/100 g protein, 6.2 g/100 g moisture, and 4.2 g/100 g ash), unsalted butter (Fonterra Co-operative Group, Auckland, New Zealand; 82.9 g/100 g lipid, 15.88 g/100 g moisture, 0.6 g/100 g protein, 0.6 g/100 g carbohydrate, and 0.02 g/100 g ash), white sugar (Samyang Co., Ltd., Seoul, Korea), salt (Daesang Co., Ltd., Seoul, Korea), and instant dry yeast (Everhealthcare Co., Ltd., Seoul, Korea) were purchased from Coupang (Seoul, Korea).

For proximate composition analysis, sulfuric acid (≥98%), boric acid, potassium sulfate, hydrochloric acid standard solution (0.1 N), sodium hydroxide solution (40% in water), and petroleum ether were purchased from Daejung Chemical & Metals Co., Ltd. (Gyeonggi-do, Korea). Sodium hydroxide was purchased from Junsei Chemical Co., Ltd. (Tokyo, Japan). Ether (≥99.5%), ethyl alcohol (≥99.9%), and copper(II) sulfate pentahydrate (≥99%) were purchased from Samchun Chemicals Co., Ltd. (Seoul, Korea). Bromocresol green methyl red solution was purchased from Sigma-Aldrich Chemical Co. (St. Louis, MO, USA). Deionized water employed in the experiments was purified using a Milli-Q system (Millipore Co., Milford, MA, USA).

### 2.2 Breadmaking process

The bread formula was developed by adjusting the water content while replacing bread flour with SPI based on the white bread recipe from the Korean Baker's License (**Table S1**). Protein-fortified bread without SCG was used as the control for comparison. The dough was prepared by substituting 1%, 3%, 5%, 10%, or 15% (*w*/w) of the combined 300 g of bread flour and SPI with SCG. This substitution range was strategically selected: lower levels (1–5%) aimed to evaluate quality maintenance and consumer sensory acceptability, while higher levels (10–15%) were incorporated to investigate the physical limits and extreme microstructural disruptions of the dough matrix upon high dietary fiber addition. Similar to the baker's percentage, the sum of bread flour, SPI, and SCG was set to 100%, and the proportion of each ingredient relative to this total is shown in **Table S2**.

Water, salt, sugar, bread flour, SPI, SCG, and yeast were mixed using the dough function of a bread maker (WBM—152S, Ohsung, Gyeongnam, Korea) at low speed for 6 min, followed by the addition of butter. The dough was then kneaded at medium speed for 12 min. After rounding the dough, it was placed in a bowl, covered with plastic wrap with holes, and allowed to undergo primary fermentation at 25 °C for 50 min. The dough was then divided into three equal parts, rounded, and covered with plastic wrap for a resting period of 15 min. The dough was shaped into loaves, placed in bread pans (9.5 × 9.5 × 9.5 cm), covered with plastic wrap, and subjected to secondary fermentation for 1 h. The loaves were baked in a preheated oven at 210 °C for 15 min, followed by baking at 185 °C for 15 min, and then cooled for 2 h. All measurements of the bread loaves were conducted at least 2 h after baking. Protein-fortified bread with SCG addition at combined flour and SPI substitution levels of 0%, 1%, 3%, 5%, 10% and 15% was designated as control, SCG1, SCG3, SCG5, SCG10, and SCG15, respectively.

### 2.3 Volume and specific volume measurement

The procedure for volume and specific volume measurements was conducted based on a method previously described in the literature (Xin et al., 2022). The volume and specific volume of bread were measured 1 h after baking using the sesame seed displacement method, which determines the volume of an object based on the weight of displaced sesame seeds and their density. To calculate the seed density, a container of known volume was filled with sesame seeds and weighed. After subtracting the weight of the empty container, the seed density was determined to be 0.614 g/cm^3^. For bread volume measurement, the bread was placed in an empty container, and sesame seeds were added to fill the remaining space. The weight of displaced seeds was calculated by subtracting the weight of the container holding both the bread and sesame seeds from the weight of sesame seeds required to fill the empty container, and then adding the weight of the bread. The bread volume was determined using Eq. (1).

(1) $$\mathrm{Volume}\;\left({\mathrm{cm}}^{3}\right)=\frac{\mathrm{Weight of displaced seeds}\;\left(g\right)}{\mathrm{Seed density}\;\left(g/cm^{3}\right)}$$

Finally, the specific volume was calculated using Eq. (2).

(2) $$\mathrm{Specific volume}\;\left(cm^{3}/g\right)=\frac{B\text{read volume}\;\left(cm^{3}\right)}{\text{Bread weight}\;\left(g\right)}$$

### 2.4 Color analysis

The color of the bread crumbs and SCG was determined 1 h after baking, using a color meter (NE-4000, Nippon Denshoku Industries Co., Ltd., Tokyo, Japan) in reflection mode. Before measurement, the instrument was calibrated with a standard white plate and zero calibration tile. The CIE L*, a*, b* system was used to evaluate color parameters, where the L* value represents lightness, ranging from 0 (black) to 100 (white). The a* value indicates the red-green axis, where positive values represent redness and negative values represent greenness. The b* value corresponds to the yellow-blue axis, with positive values indicating yellowness and negative values indicating blueness (Oh & Lee, 2024). The bread was sliced into 7-mm thick pieces, and a dedicated colorimeter container was used to extract samples from the center of each slice. The extracted samples were shaped according to the container's specifications. For each sample, measurements were taken from three randomly selected bread pieces, and the average value was recorded. The total color difference (ΔE*) represents the difference in color coordinates between the control and other samples, calculated using Eq. (3).

(3) $$\Delta E*=\sqrt{{\left(\Delta L*\right)}^{2}+{\left(\Delta a*\right)}^{2}+{\left(\Delta b*\right)}^{2}}$$

where ΔL*, Δa*, and Δb* represent the differences in color values between the sample and the control.

### 2.5 Scanning electron microscopy (SEM)

The microstructure of bread crumb was examined using SEM (CLARA LMH, TESCAN, Brno, Czech Republic). Bread samples were sliced into 1-mm-thick sections based on the procedure described by previous studies (Xin et al., 2022), freeze-dried, and subsequently coated with gold using an ion sputter coater (E-1010, Hitachi Science Systems, Japan). Imaging conditions were as follows: electron beam voltage, 5 kV; beam current, 100 pA; working distance (WD), 10 mm; magnifications, ×100 and ×1000.

### 2.6 Image analysis of bread crumb cells

The image analysis method was developed based on the procedure described by previous studies (Skendi et al., 2010; Xin et al., 2022). Bread samples were sliced to a thickness of 5 mm using an adjustable slicer. Slices were scanned at 150 dpi grey level using a scanner (HP OfficeJet Pro 8028, HP, USA) and the supporting software (HP Smart, Version 154.2.1075.0, HP), with a black paper background placed behind the scanner glass. **Fig. S1** illustrates the central area (25 × 25 mm^2^) of bread slices selected for image analysis. The scanned images were analyzed using ImageJ software (version 1.53e; National Institutes of Health [NIH] and the Laboratory for Optical and Computational Instrumentation, USA). Threshold values were manually determined using a method previously reported in the literature (Crowley et al., 2000). Eight parameters—total number of cells, average cell size, total area of cells, number of cells >4 mm^2^, number of cells <4 mm^2^, average area of cells >4 mm^2^, average area of cells <4 mm^2^, and ratio of cell area to total area—were measured by using this procedure.

### 2.7 Light microscopy

The microscopic structure of bread crumb samples was examined using a light microscope (CX31 RTSF, Olympus Corp., Tokyo, Japan) equipped with a digital microscope camera (ISH300, Tucsen, Fuzhou, China). Images were captured using TCapture software (Version 4.3.0.605; Tucsen). Bread samples were sliced into 1-mm-thick sections based on the procedure described by previous studies (Xin et al., 2022), and images of the central area of each slice were obtained at 40× magnification (4× objective lens and 10× eyepiece).

### 2.8 Texture profile analysis (TPA)

The experimental procedure for determining the texture of bread samples was designed with reference to (Mau et al., 2020). The texture profile analysis (TPA) was performed using a texture analyzer (TA-XT2, Stable Micro Systems, Surrey, UK) and the supporting software Texture Exponent 32 (Version 6.1.9.0; Stable Micro Systems). Bread cubes measuring 20 mm × 20 mm × 20 mm were sliced from the central part of the bread. The sample size was selected to match the dimensions used for sensory evaluation, ensuring consistency between instrumental and sensory analyses. TPA was conducted to measure textural parameters, including hardness, springiness, cohesiveness, gumminess, chewiness, and resilience, using a double compression test. A cylindrical probe with a diameter of 40 mm was used. The pre-test, test, and post-test speeds were all set at 1.0 mm/s. The trigger force was set at 8 g. In addition, the compression distance was set to 50% of the sample's original height, and the waiting time between the two compressions was set to 5 s.

### 2.9 Water activity (a_w_) and proximate analysis

The water activity (*a*_w_) of bread crumbs was determined at 25 °C, 1 h after baking, using a water activity meter (EZ-200, Freund Co., Tokyo, Japan). The instrument was calibrated prior to measurement using four saturated salt solutions: magnesium chloride hexahydrate (MgCl₂·6H₂O, *a*_w_ = 0.328), magnesium nitrate hexahydrate (Mg(NO₃)₂·6H₂O, *a*_w_ = 0.529), sodium chloride (NaCl, *a*_w_ = 0.753), and ammonium dihydrogen phosphate (NH₄H₂PO₄, *a*_w_ = 0.930). For sample preparation, the bread was sliced to a thickness of 7 mm, and samples were obtained by punching out random portions using a plastic sample container with a depth of 7 mm and a diameter of 3 cm.

The proximate composition of the bread samples and SCG was analyzed using a combination of the Association of Analytical Chemists (AOAC) official methods (AOAC, 2019) and other established techniques. Moisture content was measured using an infrared moisture analyzer (MB45, OHAUS, USA). The drying process was completed when the average weight loss was less than 1 mg for 60 s. Approximately 1.5 g of each sample was weighed in aluminum trays before analysis. The Kjeldahl method was applied to quantify crude protein, using a nitrogen-to-protein conversion factor of 6.25. The digestion process was carried out using a Kjeldahl digestor (MBC-12, RAYPA, Spain), followed by distillation with a distiller (DNP-1500 MP, RAYPA). Crude fat content was analyzed using the AOAC Official Method 945.16, based on Soxhlet extraction with petroleum ether. Crude ash content was determined after preliminary drying, followed by combustion in a muffle furnace (MF-05, HYSC, Korea) at 600 °C until a constant final weight was achieved. Crude fiber content was measured using the AOAC Official Method 978.10. Carbohydrate content was estimated by difference, subtracting the measured values of moisture, crude protein, crude fat, and crude ash from 100. Results were expressed as grams per 100 g of the sample.

### 2.10 X-ray diffraction (XRD) analysis

XRD analysis was conducted using an X-ray diffractometer (Ultima IV, Rigaku, Japan) to determine the effect of SCG addition on the crystallinity of bread crumb. Bread crumb samples were freeze-dried, ground into a fine powder, and used for the analysis. The measurement conditions were as follows: scanning scan axis, 2θ/θ; scanning type, continuous scanning; X-ray source, Cu-Kα radiation (λ = 1.5409 Å) operated at 40 kV and 30 mA; divergence slit, 2/3°; divergence height limiting slit (DivH.L slit), 10 mm; scatter slit, 2/3°; receiving slit, 0.3 mm. The diffraction data were collected over a 2θ range from 5° to 40°, with a step size of 0.02° and a scan speed of 1°/min. Each sample was analyzed in duplicate. The scattered intensities from the crystalline and amorphous phases were integrated using the PDXL2 software (Rigaku), and the RC was calculated using Eq. (4).

(4) $$\mathrm{RC}\;\left(\%\right)=\frac{\text{Area of crystalline peaks}}{\text{Area of crystalline peaks}+\text{Area of amorphous region}}\times 100\%$$

### 2.11 Sensory evaluation

Sensory evaluation was conducted with 30 untrained panelists recruited from Dongguk University students (9 males and 21 females; age range: 22–43 years; mean age: 26 years). All participants were confirmed to have no allergies to bread flour, soy, or coffee. Prior to participation, full details of the study were explained to all volunteers, written informed consent was obtained, and participants were informed that all data would be treated anonymously and confidentially and that they had the right to withdraw at any time. The sensory evaluation procedure was reviewed and approved by the Dongguk University Institutional Review Board (DUIRB2025-04-06).

For sample preparation, six types of bread samples were prepared and baked 1 day before serving. After cooling to room temperature, the bread samples were stored in zipper-sealed plastic bags. Samples (2 cm × 2 cm × 2 cm) were cut from the center of each bread slice and served alongside a whole bread piece on white plates. To minimize sensory fatigue and carryover effects, particularly due to the bitterness of SCG, samples were evaluated in the following order: control, SCG1, SCG3, SCG5, SCG10, and SCG15. In terms of sensory attributes, the hedonic evaluation assessed appearance, aroma, taste, texture, and overall acceptability. Additionally, specific attribute intensities, including moistness, coffee aroma, sweetness, bitterness, and softness, were evaluated. A 9-point hedonic scale was used (1 = dislike extremely, 2 = dislike very much, 3 = dislike moderately, 4 = dislike slightly, 5 = neither like nor dislike, 6 = like slightly, 7 = like moderately, 8 = like very much, 9 = like extremely). During the evaluation, panelists were instructed to cleanse their palates between samples with water and plain crackers. All evaluations were conducted in a quiet and comfortable sensory evaluation laboratory equipped with white lighting. During the sensory evaluation, panelists were also encouraged to provide optional comments regarding their impressions of each sample.

### 2.12 Statistical analysis

Statistical analyses were conducted using IBM SPSS Statistics 27 (IBM, Armonk, NY, USA). Except for XRD measurements and sensory evaluation, all experiments were performed in triplicate (*n* = 3). Results were expressed as mean ± standard deviation (SD). One-way analysis of variance (ANOVA) was used to detect significant differences among the sample groups, and Tukey's honestly significant difference (HSD) test was applied for post hoc comparisons at a significance level of *p* < 0.05.

## 3 Results and discussion

### 3.1 Specific volume and color of bread

**Fig. 1:**
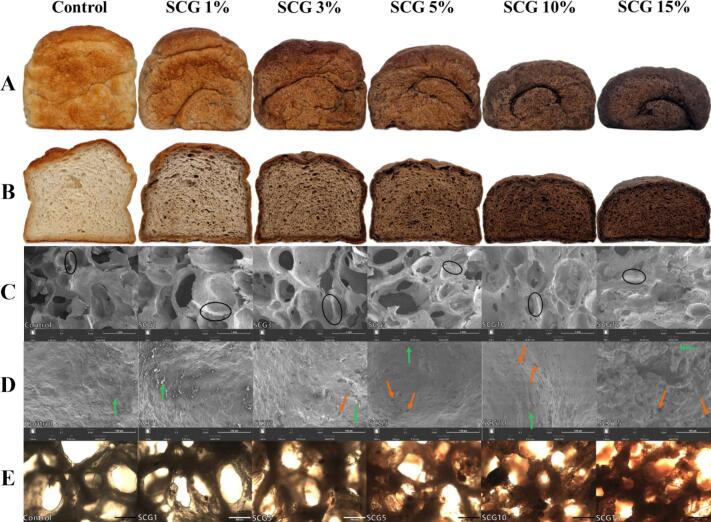
The appearance (A), crumb cross-section (B), scanning electron microscopy at 100× (C) and 1000× (D), optical microscopy images (E) of protein-fortified breads with varying levels of SCG (Control, SCG1, SCG3, SCG5, SCG10, and SCG15). Breads were prepared by replacing 0%, 1%, 3%, 5%, 10%, and 15% (*w*/w) of bread flour with SCG, respectively. Black ovals indicate cell walls, green arrows indicate starch or starch granules, and orange arrows indicate discontinuous gluten networks. The scale bars represent 1 mm in (C), 100 μm in (D), and 500 μm in (E). (For interpretation of the references to color in this figure legend, the reader is referred to the web version of this article.)

As shown in Fig. 1A, the volume of bread decreased with increasing SCG content. This is also quantitatively confirmed by the data presented in Table 1. Compared to the control, the specific volumes of SCG1, SCG3, SCG5, SCG10, and SCG15 decreased by 2.05%, 6.16%, 15.84%, 35.78%, and 40.76%, respectively. Significant reductions (*p* < 0.05) were observed in SCG3, SCG5, SCG10, and SCG15. This result is consistent with previous findings, which show that increased levels of fiber-rich ingredients significantly reduce the specific volume of bread (Xin et al., 2022). The reduction in specific volume can be attributed to the decrease in the dough's gluten-forming capacity; as the amount of bread flour decreased while the dough weight remained constant. As the flour content declined; the amount of gluten that could form also decreased; leading to reduced dough elasticity and a lower capacity to retain the gas produced during fermentation. Proteins in SCG lack gluten-forming ability. These proteins may interfere with gluten network formation by disrupting gliadin–glutenin interactions. This disruption results in decreased dough extensibility and limited gas retention during fermentation (Zhang et al., 2023). According to a previous study; fiber could disrupt the gluten network during baking; reducing the water-binding capacity and gas retention ability of gluten (Wang et al., 2021). The presence of SPI could have diluted the gluten network; thereby limiting the extent of volume expansion (Zhou et al., 2018). In addition; the high water absorption capacity of SCG has been reported (Murthy & Naidu, 2012). The dietary fiber in SCG likely competes with bread flour for water; reducing the amount of water available for gluten hydration and dough expansion (Verbeke et al., 2024). This significant reduction in specific volume is highly consistent with recent studies on SCG-supplemented bread. As reported by previous studies; the physical interference and high water absorption of SCG insoluble dietary fibers universally dilute the gluten matrix and restrict volume expansion during baking (Ahanchi et al., 2024; Cacak-Pietrzak et al., 2024).

**Table 1:** Physicochemical and sensory properties of protein-fortified bread formulated with varying levels of SCG.

Quality characteristics	Control	SCG 1	SCG 3	SCG 5	SCG 10	SCG 15
Volume (cm^3^)	601.88 ± 4.39^d^	584.86 ± 21.03^cd^	557.81 ± 15.02^c^	505.58 ± 14.39^b^	386.55 ± 5.77^a^	357.34 ± 9.10^a^
Specific volume (cm^3^/g)	3.41 ± 0.02^d^	3.34 ± 0.12^cd^	3.20 ± 0.06^c^	2.87 ± 0.08^b^	2.19 ± 0.03^a^	2.02 ± 0.04^a^
Moisture (g/100 g)	50.10 ± 0.51^a^	49.54 ± 0.41^a^	50.00 ± 0.93^a^	50.43 ± 0.55^a^	50.10 ± 0.11^a^	48.92 ± 1.15^a^
Carbohydrate (g/100 g)	34.95 ± 0.80^b^	35.05 ± 0.35^b^	34.27 ± 0.79^ab^	33.49 ± 0.65^ab^	32.43 ± 0.32^ab^	33.41 ± 1.07^ab^
Crude protein (g/100 g)	11.07 ± 0.19^a^	11.18 ± 0.09^a^	11.25 ± 0.09^ab^	11.30 ± 0.36^ab^	11.71 ± 0.18^bc^	11.86 ± 0.11^c^
Crude fat (g/100 g)	2.67 ± 0.56^a^	2.98 ± 0.05^a^	3.20 ± 0.06^a^	3.46 ± 0.28^a^	4.42 ± 0.37^b^	4.48 ± 0.03^b^
Crude ash (g/100 g)	1.21 ± 0.07^a^	1.24 ± 0.06^a^	1.28 ± 0.05^a^	1.31 ± 0.02^a^	1.33 ± 0.02^a^	1.34 ± 0.11^a^
Crude fiber (g/100 g)	0.07 ± 0.01^a^	0.29 ± 0.36^a^	0.39 ± 0.04^a^	0.97 ± 0.08^b^	2.05 ± 0.13^c^	2.97 ± 0.15^d^
Water activity	0.980 ± 0.000^c^	0.963 ± 0.012^abc^	0.947 ± 0.006^a^	0.957 ± 0.006^ab^	0.967 ± 0.006^bc^	0.963 ± 0.006^abc^
Relative crystallinity (%)	34.30 ± 1.48^a^	33.71 ± 0.61^a^	33.73 ± 0.81^a^	34.65 ± 2.09^a^	34.07 ± 0.88^a^	35.35 ± 1.32^a^
**Crumb color value**						
L*	67.66 ± 0.48^f^	49.42 ± 2.55^e^	35.35 ± 0.45^d^	26.81 ± 0.60^c^	19.85 ± 0.19^b^	16.48 ± 0.74^a^
a*	2.33 ± 0.27^a^	4.83 ± 0.37^b^	6.17 ± 0.45^c^	6.55 ± 0.22^c^	5.78 ± 0.27^c^	4.80 ± 0.11^b^
b*	22.79 ± 0.20^f^	17.25 ± 0.39^e^	15.49 ± 0.17^d^	13.39 ± 0.15^c^	9.39 ± 0.11^b^	7.50 ± 0.34^a^
∆E		19.26 ± 2.80^a^	33.58 ± 1.06^b^	42.13 ± 0.44^c^	49.77 ± 0.60^d^	53.48 ± 0.70^e^
**Sensory attributes**						
Appearance	7.90 ± 1.18^c^	6.90 ± 1.45^bc^	7.00 ± 1.36^bc^	6.43 ± 1.57^b^	4.70 ± 1.64^a^	3.80 ± 1.92^a^
Aroma	6.73 ± 1.36^b^	6.80 ± 1.61^b^	6.63 ± 1.56^b^	6.60 ± 1.35^ab^	6.17 ± 1.74^ab^	5.40 ± 2.14^a^
Taste	6.63 ± 1.61^c^	6.33 ± 1.52^c^	6.27 ± 1.62^c^	5.87 ± 1.55^bc^	4.87 ± 1.53^ab^	4.00 ± 1.70^a^
Texture	6.63 ± 1.75^b^	6.20 ± 1.67^b^	6.33 ± 1.35^b^	6.40 ± 1.65^b^	5.53 ± 1.91^ab^	4.87 ± 2.05^a^
Overall acceptability	6.73 ± 1.60^b^	6.37 ± 1.59^b^	6.33 ± 1.63^b^	6.20 ± 1.40^b^	4.83 ± 1.68^a^	3.90 ± 1.56^a^
Moistness	6.50 ± 1.68^b^	5.57 ± 1.76^ab^	5.57 ± 1.68^ab^	5.70 ± 1.74^ab^	5.40 ± 1.79^ab^	4.47 ± 1.81^a^
Coffee aroma	1.10 ± 0.40^a^	2.83 ± 0.95^b^	4.53 ± 1.17^c^	5.57 ± 1.30^d^	6.77 ± 1.17^e^	7.40 ± 1.52^e^
Sweetness	5.10 ± 1.54^d^	4.43 ± 1.25^cd^	4.10 ± 1.16^bc^	3.77 ± 1.14^bc^	3.20 ± 1.24^ab^	2.70 ± 1.37^a^
Bitterness	1.77 ± 1.33^a^	2.63 ± 1.59^ab^	3.33 ± 1.58^bc^	4.10 ± 1.67^c^	6.00 ± 1.53^d^	7.20 ± 1.49^e^
Softness	6.70 ± 1.74^c^	5.57 ± 1.76^abc^	5.93 ± 1.36^bc^	5.70 ± 1.44^bc^	4.97 ± 1.77^ab^	4.30 ± 2.09^a^

All values are shown as mean ± S.D. (standard deviation)

Lowercase letters series (“a–f”) in the row column indicate significant differences (Tukey's test, p < 0.05).

The color values of the crumb of bread produced with SCG are presented in Table 1. Bread color is one of the most important factors influencing consumer purchasing decisions (Gellynck et al., 2009). As observed in Fig. 1A; as the amount of SCG increased; the bread color approached that of SCG; and the L* and b* values significantly decreased (*p* < 0.05). These color changes may give consumers the expectation of a coffee-like flavor. Dark or brown tones reminiscent of chocolate are also attractive to consumers; which could broaden consumer choices (López-Silva & García-Valle, 2024). The changes in bread color were attributed to the incorporation of SCG and its pigments; which diffused throughout the dough during the mixing process. Regarding the a* value; it initially increased with the addition of SCG and then decreased. The initial increase and subsequent decrease in the a* value could be explained by the presence of brownish pigments in SCG; such as melanoidins and polyphenols; which may have imparted a slightly reddish-brown hue to the bread at low addition levels. However; as the amount of SCG increased; the visibility of darker tones associated with Maillard reactions may have become more pronounced; potentially leading to a decrease in chroma and consequently lowering the a* value. SCG15 displayed higher L*; a*; and b* values and a lower ∆E* value compared to SCG. The Maillard reaction processes diminish bread's red-yellow tones while darkening its overall appearance. These modifications result in more noticeable color differences. This pronounced decrease in L* and corresponding shifts in a* and b* values closely parallel the observations made in other bakery products fortified with coffee by-products (Cacak-Pietrzak et al., 2024; Rizkaprilisa et al., 2023), confirming that the natural dark brown pigments of SCG fundamentally alter the visual characteristics of the baked goods.

### 3.2 Effect of SCG addition on the microstructure of bread

SEM was employed to investigate the microstructural changes in bread crumb and elucidate the reasons behind the observed reduction in bread volume. SEM images at magnifications of 100× and 1000× are presented in Fig. 1C and Fig. 1D, respectively. As shown in Fig. 1C, the size of crumb cells gradually decreased as the SCG level increased from 0% (control) to 15% (SCG15), and smaller cells and pores became more numerous, indicating an increased porosity. The formation of smaller and more numerous crumb cells may result from dietary fibers disrupting the dough foam structure during fermentation, creating additional pores (W. Han et al., 2017). Simultaneously; cell walls became thicker with increasing SCG content; thus reducing the proportion of area occupied by cells and leading to higher crumb density and decreased specific volume. These changes in microstructure are consistent with previous findings; which demonstrated a denser crumb and lower loaf volume when the level of wheat flour replacement reached 20% and 25% lychee pulp dietary fiber (Xin et al., 2022). The control; SCG1; and SCG3 samples showed relatively uniform cell sizes; with balanced distributions of larger and smaller cells; representing an ideal crumb structure. In the remaining treatments; the cell distribution became uneven; and in SCG15; regions showing a clumping phenomenon due to impaired gluten interactions were evident. Similar observations were reported by (Ni et al., 2020).

In Fig. 1D, gelatinized starch granules embedded within the crumb structure were clearly visible. The control and SCG1 samples exhibited evenly distributed gluten matrices with smooth textures. The SCG15 sample exhibited severe structural deterioration, characterized by starch granules that were poorly integrated into the gluten matrix and existed primarily as isolated or clustered aggregates. Discontinuous gluten networks, as evident in indentations or voids, increased notably with higher levels of SCG addition. As the discontinuities in the gluten network increased, a greater number of cracks and voids formed within the crumb structure. These defects impaired the crumb's ability to retain fermentation gases, leading to gas leakage through the cracks and voids, which ultimately limited bread volume expansion during baking (Djordjević et al., 2022).

### 3.3 Bread crumb structure

**Fig. 2:**
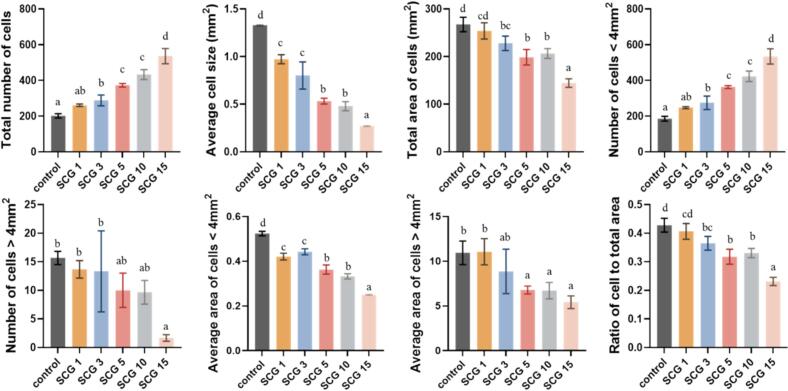
Crumb cell characteristics of protein-fortified bread with SCG (Control, SCG1, SCG3, SCG5, SCG10, and SCG15) prepared by replacing 0%, 1%, 3%, 5%, 10%, and 15% (w/w) of bread flour with SCG, respectively.

Cross-sectional images of bread samples are presented in Fig. 1B, and quantitative data on cell characteristics obtained through image analysis are summarized in Fig. 2. The total number of cells increased as the SCG content increased. Bread volume and cross-sectional area decreased, which means that more cells were packed into a smaller space, resulting in smaller average cell sizes. The image analysis confirmed this relationship, showing a progressive decrease in average cell size with increasing SCG concentration. All loaves were prepared using the same dough mass, so a smaller total area occupied by cells should theoretically result in a smaller bread volume. This theoretical assumption was validated by the experimental data, which showed a strong positive correlation between bread volume and total cell area. Cells were further classified into two groups based on size (<4 and > 4 mm^2^), and their respective characteristics were analyzed. As SCG addition increased, the number of smaller cells (<4 mm^2^) increased, whereas the number of larger cells (>4 mm^2^) decreased. Meanwhile, the average cell area decreased for both groups. The increase in the number of smaller cells accompanied by a reduction in their individual sizes indicates an increase in crumb fineness, which is closely related to the textural perception when chewing bread (Skendi et al., 2010). Increased crumb fineness contributes to a denser and tighter crumb structure.

The ratio of cell area to total area, calculated by dividing the total area of cells by the total cross-sectional area of the image (625 mm^2^), represents the crumb's void fraction. This void fraction decreased with increasing SCG levels, a trend also visually confirmed in the SEM images (Section 3.2). The observed reduction in the void fraction can be attributed to the thickening of cell walls and a decrease in cell size, resulting in a reduction of the proportion of void space relative to the total crumb area. These results suggest that the crumb structure became denser with higher SCG incorporation. Previous studies have reported that higher crumb porosity is associated with softer texture and greater consumer acceptability in bread (Angioloni & Collar, 2009; Zghal et al., 2002). Although no universal standard exists for optimal crumb porosity, a higher cell area-to-total area ratio is generally associated with softer crumb and better bread quality. The decreased void fraction observed in SCG-substituted breads may have negatively affected texture by contributing to increased hardness and density. Overmixing bread dough can increase the number of cells and simultaneously decrease the void fraction compared to optimally mixed dough (Crowley et al., 2000). The addition of SCG may have reduced the optimal mixing time. If mixed for the same duration as the control, these samples could have been overmixed, resulting in excessive cell formation and a lower cell area-to-total area ratio.

In Fig. 1E, cross-sectional images of bread samples observed by light microscopy visually demonstrate the cellular structures of bread crumbs. As the SCG levels increased, the crumb color gradually became darker, approaching the brownish shade of SCG, and particles of SCG were increasingly observed trapped within the cell walls. This observation indicates that SCG particles may disrupt the gluten network structure. Additionally, as the SCG content increased, the cells became smaller and more numerous, and the previously smooth and continuous network structures transitioned into a rougher and more irregular morphology, reflecting an unstable crumb structure.

### 3.4 Texture properties of bread crumb

**Fig. 3:**
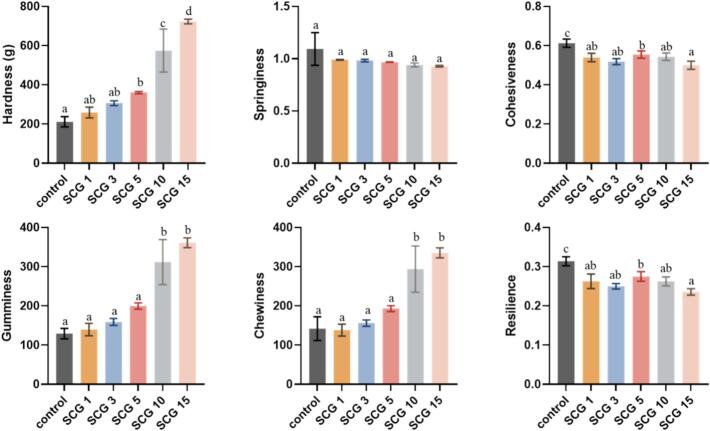
Texture characteristics of bread crumb (Control, SCG1, SCG3, SCG5, SCG10, and SCG15) prepared by replacing 0%, 1%, 3%, 5%, 10%, and 15% (w/w) of bread flour with SCG, respectively.

TPA was conducted to evaluate how the structural changes observed in 3.2, 3.3 influenced bread texture properties. Bread crumb hardness significantly increased upon SCG addition (Fig. 3, *p* < 0.05). An inverse relationship between void fraction and crumb hardness has been reported, supporting the present findings (Zghal et al., 2002). Samples SCG10 and SCG15; exhibiting lower ratios of cell area-to-total-area (lower void fractions); presented notably higher hardness. This can be attributed to the increased crumb density resulting from reduced bread volume; as demonstrated in previous sections. Breads with smaller and densely packed crumb cells exhibit greater resistance to mastication. The presence of nonpolar lipids from incorporated ingredients may interact with starch and contribute to increased crumb hardness (Lu et al., 2016). It is plausible that nonpolar lipids from SCG contributed to the increased hardness.

Cohesiveness, which reflects the structural integrity and internal binding strength of bread crumb, decreased with increasing SCG content. Decreased cohesiveness has been found to correlate with a higher tendency for crumb fragmentation (Onyango et al., 2011). The inconsistent trend in cohesiveness observed across SCG samples may be partially attributed to the fluctuation in RC; specifically the lack of significant differences; as determined by XRD analysis (Table 1). SCG15 exhibited the lowest cohesiveness; possibly because the substantial SCG content reduced the crumb moisture; leading to a more crumbly and fragile texture. Gumminess; reflecting the sticky mouthfeel upon mastication; is calculated as the product of hardness and cohesiveness. Lower gumminess implies a softer texture; making the bread easier to chew. Although cohesiveness values showed relatively minor variations among samples; the substantial increase in hardness with increasing SCG levels resulted in a pronounced increase in gumminess. Chewiness represents the energy required to chew bread until it is suitable for swallowing. Harder textures inherently require greater chewing effort. As previously discussed in Section 3.3; the disrupted moisture absorption and gluten formation caused by SCG addition resulted in denser crumb structures; consequently increasing instrumental chewiness. These textural changes align well with other recent literature; which confirmed that SCG addition substantially increases bread firmness and chewiness due to the dense packing of crumb cells and reduced porosity (Cacak-Pietrzak et al., 2024). Similar texture profiles; characterized by increased density and hardness; have also been reported in SCG-enriched cookies (Papageorgiou et al., 2024). Resilience represents the crumb's ability to recover after compression. The observed reduction in resilience is likely associated with weakened elasticity of the gluten network, impairing the crumb's elastic recovery capability.

### 3.5 Physicochemical characteristics of bread

The proximate composition data of protein-fortified bread supplemented with SCG are presented in Table 1. The bread's proximate composition followed the order of moisture, carbohydrates, crude protein, crude fat, and crude ash content. As the SCG content increased, the levels of crude protein, crude fat, and crude fiber significantly increased (*p* < 0.05). Specifically, the SCG15 sample, which exhibited the most pronounced differences, showed increases of 7.1%, 67.8% (from 2.67 to 4.48 g/100 g), 10.7%, and 4142.9% (from 0.07 to 2.97 g/100 g) in crude protein, crude fat, ash, and crude fiber, respectively, compared to the control. These differences were attributed to the composition of SCG itself, which has 89.5% lower moisture and 12.2% lower carbohydrate contents compared to bread flour. In contrast, crude protein, crude fat, and ash contents in SCG were higher by 48.1%, 1047.9%, and 48.1%, respectively. Moreover, SCG contains 39.4 g/100 g of crude fiber, while bread flour does not contain any. These significant enhancements in the nutritional profile, particularly the remarkable increases in dietary fiber and ash contents, are highly consistent with results described by previous studies. The incorporation of coffee by-products has been widely reported to effectively fortify the bread matrix with dietary fibers and essential minerals (Ahanchi et al., 2024; Rizkaprilisa et al., 2023). Moisture content did not differ significantly among the samples, but it was lowest in SCG15. This may be due to the inherently lower moisture content of SCG compared to bread flour, despite the total amount of water added during dough preparation remaining constant. The lower bread volume of SCG15 could also be attributed to its lower moisture content, as insufficient water availability may have hindered proper gluten formation. Additionally, because lower moisture content typically leads to a harder crumb texture, this may explain the increased hardness of SCG15 bread. Carbohydrate content decreased as the SCG addition increased, although the differences were not statistically significant. This may be because dietary fiber, which is abundant in SCG, is included in the total carbohydrate content. Fats present in SCG can impart desirable flavor characteristics in baked goods (López-Silva & García-Valle, 2024). These lipids may also enhance the sensory profile of the protein-fortified bread supplemented with SCG. Moreover; SCG contains a high proportion of linoleic acid; an essential fatty acid comprising approximately 43% of the total fat content; offering additional nutritional benefits. Crude fiber content surpassed the ash content in the SCG10 and SCG15 bread samples. Compared to the control; SCG10 and SCG15 showed dramatic increases of approximately 28- and 41-fold; respectively; due to the extremely low fiber content typically found in conventional bread formulations. The rich polyphenol and dietary fiber content of SCG may contribute to reduced postprandial glycemic response by decreasing α-amylase activity and hindering starch digestion (Liu et al., 2022).

**Fig. 4:**
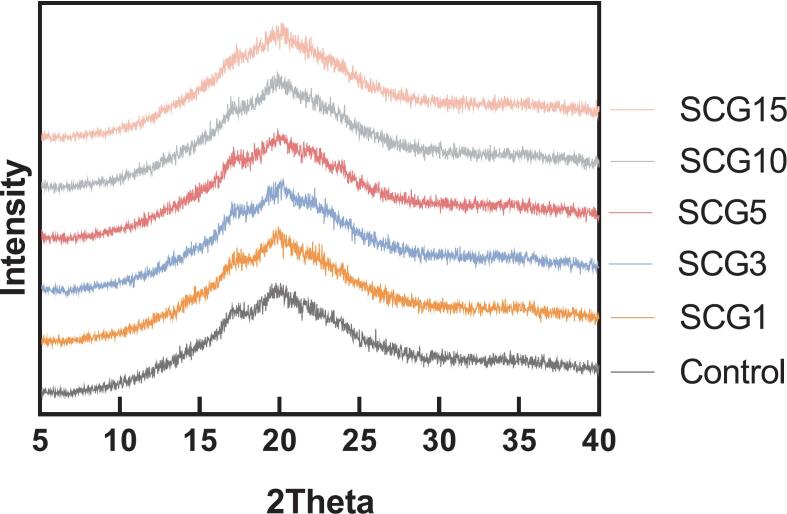
X-ray diffractograms of protein-fortified bread with SCG; Control, SCG1, SCG3, SCG5, SCG10, and SCG15: bread made by replacing 0%, 1%, 3%, 5%, 10%, and 15% (w/w) of bread flour with spent coffee grounds, respectively.

To investigate whether the addition of SCG affects the crystallinity of bread, XRD analysis was conducted. Fig. 4 presents the X-ray diffractograms of the samples, all of which exhibited a common peak at around 20°. This peak represents a V-type diffraction pattern generally attributed to the formation of amylose-lipid complexes (Salgado-Cruz et al., 2017). In this study, these complexes might have originated from interactions among bread flour components, butter lipids, and the lipids contained in SCG. Foods with higher crystallinity typically possess greater structural stability and are less susceptible to external mechanical stress (Xu et al., 2023). Previous studies have reported that increasing the amount of dietary fiber-rich ingredients generally reduces the intensity of crystallinity in bread formulations (S. Han et al., 2024; Santos et al., 2024; Xin et al., 2022). However, as indicated in Table 1, no significant differences in RC were observed among the samples. Therefore, unlike other dietary fiber sources, SCG did not decrease the crystallinity of bread even at its highest addition levels examined, suggesting an advantageous property of SCG in maintaining structural integrity.

### 3.6 Sensory evaluation

**Fig. 5:**
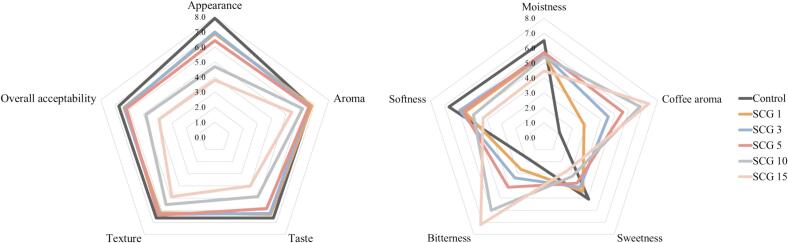
Sensory attributes of protein-fortified bread crumbs with different levels of spent coffee grounds; Control, SCG1, SCG3, SCG5, SCG10, and SCG15: bread made by replacing 0%, 1%, 3%, 5%, 10%, and 15% (w/w) of bread flour with spent coffee grounds, respectively.

Consumer acceptance is a critical factor in the commercialization of novel food products. A sensory evaluation was conducted to assess the acceptability of bread supplemented with SCG. The results of the sensory preference and attribute analyses for protein-fortified bread are presented in Table 1 and Fig. 5. Attributes intuitively related to SCG, such as coffee aroma and bitterness intensity, significantly increased as the level of SCG addition increased (*p* < 0.05). However, the SCG1, SCG3, and SCG5 bread samples showed no significant differences in aroma, taste, texture, overall acceptability, moistness, and softness compared to the control. For appearance, SCG10 and SCG15 were rated significantly lower than the other samples due to their excessively dark crumb, which resembled a burnt surface, and markedly reduced loaf volume, which diminished their resemblance to typical bread. These samples received the lowest scores for overall acceptability because of the persistent perception of coarse particles, pronounced bitterness, astringency, and burnt aroma, all of which remained perceptible during oral evaluation.

Among the panelists, 33%, 33%, and 30% preferred the SCG1, SCG3, and SCG5 bread samples, respectively, over the control. Many panelists who preferred SCG-supplemented samples reported that the control bread was relatively bland. In contrast, bread containing SCG offered a more appealing coffee aroma, making it less likely to become monotonous. Notably, even individuals who disliked or were indifferent toward coffee expressed a preference for the SCG1 sample, indicating that incorporating SCG can introduce new, appealing flavors that enhance the overall acceptability of the bread. Some panelists expressed willingness to purchase the product, suggesting its potential market acceptability. The significantly enhanced coffee aroma scores can be supported by the well-documented volatile profile of SCG. A rich profile of volatile compounds, predominantly pyrazines, furans, and aldehydes, which are universally recognized as the key contributors to the desirable roasted coffee flavor, has been demonstrated to be retained in roasted SCG, as described by a previous study (Andrade et al., 2022b). Furthermore; the excellent sensory balance and rich aroma of SCG-supplemented bread have been verified through expert sensory evaluations described in a previous study (Bulkaini et al., 2024), strongly supporting the high hedonic scores observed in our formulations.

Hardness has been identified as the most important textural attribute influencing consumer acceptance of bread (Ni et al., 2020). Although the SCG15 sample exhibited the highest instrumental hardness, sensory evaluation comments revealed that panelists who favored drier and firmer bread textures found the SCG15 sample particularly appealing. Thus, deliberately increasing the level of SCG addition could be a viable strategy for targeting consumers who prefer a firmer bread texture. These findings indicate that when added at appropriate levels, SCG can improve consumer acceptance of bread by enhancing the flavor diversity, highlighting its promising commercial potential as a functional food ingredient.

## 4 Conclusion

This study investigated the impact of SCG incorporation on the quality characteristics, microstructure, physicochemical properties, and sensory acceptability of protein-fortified bread. The addition of SCG improved the nutritional profile of bread by increasing its protein and dietary fiber contents, thereby compensating for the carbohydrate-dominant nature of conventional bread. However, excessive incorporation led to the formation of a discontinuous gluten network, resulting in smaller cells, reduced loaf volume, and a firmer texture. Sensory evaluation indicated that breads containing 1–5% SCG showed similar overall acceptability to the control and received positive comments from some panelists due to their coffee aroma and flavor complexity. Based on these findings, the incorporation of SCG at 3–5% is recommended for optimizing nutritional value, structural quality, and consumer acceptance. In addition to its nutritional and sustainability advantages, the incorporation of SCG imparts a desirable roasted coffee aroma, as evidenced by our sensory evaluation. This suggests that SCG can serve as a viable natural alternative to synthetic flavor additives in bakery formulations, potentially increasing consumer appeal while reducing formulation costs. While this study focused on the microstructural, physical, and macroscopic sensory changes, the precise chemical profiling of volatile compounds and the potential biological interactions between SCG components and yeast activity remain to be elucidated. Further research investigating the specific flavor chemistry and yeast fermentation kinetics would provide a more comprehensive understanding of these complex systems. This study supports the potential of SCG as a sustainable, upcycled ingredient for value-added bakery applications.

## CRediT authorship contribution statement

**Daesik Jo:** Writing – original draft, Investigation, Formal analysis. **Woojin Na:** Methodology, Formal analysis. **Yujin Wang:** Validation, Software. **Kwang-Geun Lee:** Writing – original draft, Funding acquisition, Conceptualization.

## Declaration of generative AI and AI-assisted technologies in the writing process

During the preparation of this work the author(s) used ChatGPT in order to improve language and readability, and to assist in identifying relevant literature. After using this tool/service, the author(s) reviewed and edited the content as needed and took full responsibility for the content of the publication.

## Declaration of competing interest

The authors declare that they have no known competing financial interests or personal relationships that could have appeared to influence the work reported in this paper.

## Data Availability

Data will be made available on request.
