# Supplementary material for: Effect of spent coffee grounds addition on the microstructure and quality characteristics of protein-fortified bread
Source: Food Chem X. 2026 Mar 27;35:103796. doi: 10.1016/j.fochx.2026.103796 (PMC13062548; doi:10.1016/j.fochx.2026.103796)
Supplement: Supplementary file 1 — Supplementary material [file mmc1.docx]

**Table S1**. The original recipe for white pan bread according to the Korean Baker’s License standard.

| **Ingredients** | **Baker’s Percentage (%)** | **Weight (g)** |
| --- | --- | --- |
| Bread flour | 100 | 1200 |
| Water | 63 | 756 |
| Fresh yeast | 5 | 60 |
| Sugar | 5 | 60 |
| Shortening | 4 | 48 |
| Skim milk powder | 3 | 36 |
| Bread improver | 2 | 24 |
| Salt | 1.8 | 21.6 |
| **Total** | **183.8** | **2205.6** |

*Note:* This recipe is the official standard provided by the Human Resources Development Service of Korea (Q-Net). In the present study, shortening was replaced with unsalted butter, and the bread flour was partially substituted with soy protein isolate (SPI) and spent coffee grounds (SCG).

*Official link to the Korean Baker’s License webpage:* [https://q-net.or.kr/crf005.do?id=crf00503&jmCd=7893#](https://q-net.or.kr/crf005.do?id=crf00503&jmCd=7893)

**Table S2**. Formulation of protein-fortified bread with partial replacement of bread flour by spent coffee grounds.

| **Ingredient (%)** | **Control** | **SCG1** | **SCG3** | **SCG5** | **SCG10** | **SCG15** |
| --- | --- | --- | --- | --- | --- | --- |
| Bread flour | 90 | 89 | 87 | 85 | 80 | 75 |
| Soy protein isolate | 10 | 10 | 10 | 10 | 10 | 10 |
| Spent coffee grounds | 0 | 1 | 3 | 5 | 10 | 15 |
| Water | 88 | 88 | 88 | 88 | 88 | 88 |
| Unsalted butter | 6 | 6 | 6 | 6 | 6 | 6 |
| Sugar | 6 | 6 | 6 | 6 | 6 | 6 |
| Salt | 2 | 2 | 2 | 2 | 2 | 2 |
| Instant yeast | 1.8 | 1.8 | 1.8 | 1.8 | 1.8 | 1.8 |
| Total | 203.8 | 203.8 | 203.8 | 203.8 | 203.8 | 203.8 |

Control, SCG1, SCG3, SCG5, SCG10, and SCG15: bread samples prepared by replacing bread flour with spent coffee grounds at levels of 0%, 1%, 3%, 5%, 10%, and 15% (w/w), respectively


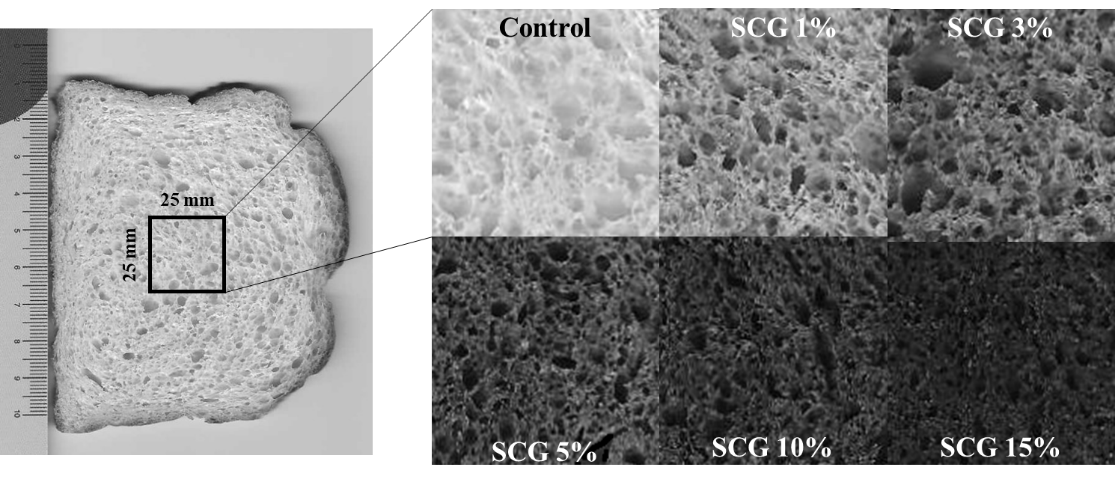


**Fig. S1**. Representative scanned image of bread crumb and magnified images (25 mm × 25 mm) of samples containing different SCG concentrations (Control, SCG 1%, SCG 3%, SCG 5%, SCG 10%, and SCG 15%).
